# A thyroid storm patient with protracted disturbance of consciousness and reversible lesion in the splenium of corpus callosum: A case report

**DOI:** 10.1097/MD.0000000000009949

**Published:** 2018-02-16

**Authors:** Chihiro Namatame, Tomohiro Sonoo, Kazutaka Fukushima, Hiromu Naraba, Hideki Hashimoto, Kensuke Nakamura

**Affiliations:** Department of Emergency and Critical Care Medicine, Hitachi General Hospital, Johnan-cho, Hitachi, Ibaraki, Japan.

**Keywords:** Hashimoto's encephalopathy, steroid-responsive encephalopathy with autoimmune thyroiditis, thyroid storm-associated encephalopathy

## Abstract

**Rationale:**

Various neurological manifestations are observed in thyroid storm patients but protracted disturbance of consciousness is rare.

**Patient concerns:**

A 58-year-old male was admitted to our hospital after a traffic accident.

**Diagnoses:**

Although awake on arrival, he fell into coma after admission. Based on the clinical symptoms and hyperthyroidism, the patient was diagnosed with thyroid storm (TS).

**Interventions:**

Even after improvement of hyperthyroidism, disturbance of consciousness was protracted. Considering the possibility of immune-related etiology, methylprednisolone pulse was started.

**Outcomes:**

His consciousness level improved over a 3-month period, and he became able to walk with some assistance after 6 months.

**Lessons:**

His condition was atypical of TS-associated encephalopathy because of the long clinical course. Reversible splenial lesion was visible using brain imaging. In some cases of TS, disturbance of consciousness can be protracted for several months, but it is reversible. Therefore, it is necessary to judge the long-term neurological outcome carefully.

## 1 Background

Thyroid storm (TS), acute thyrotoxicosis originating from poorly controlled thyroid disease, is a life-threatening condition that usually requires intensive care. Various neurological manifestations are observed in 28.5% to 72% of TS cases,^[1–3]^ but few patients fall into a coma. In most cases, neurological manifestations improve rapidly after normalization of thyroid function.^[1]^

Steroid-responsive encephalopathy with autoimmune thyroiditis (SREAT), also called Hashimoto's encephalopathy, is characterized by antithyroid antibody-positive encephalopathy developing nonspecific neurological manifestations, and distinct from TS-associated manifestation. In most cases of SREAT, the thyroid function is not associated with disease severity. Brain imaging is normal. Although this condition's pathophysiological mechanism has not been clarified, most cases of SREAT have been reported as showing good response to corticosteroid administration.^[4]^

Herein, we present a report of a patient with severe TS accompanied by disturbance of consciousness protracted for more than 1 month. The disturbance of consciousness improved slowly after corticosteroid therapy.

## 2 Case presentation

A 58-year-old male with no known past medication history was transported by ambulance to our hospital after a traffic accident. His lower limbs had been injured by compression between the truck's steering column and seat. After he was rescued 20 minutes following the accident, he was transported by ambulance to our hospital. At arrival, he was alert and oriented.

The following were found on hospital arrival: 180 mm Hg systolic blood pressure; 204/min heart rate; 90% oxygen saturation (with a 10-L oxygen reservoir mask); 36.8°C body temperature; and Glasgow coma scale (GCS), E4V4M6 with restlessness. Results of focused assessment sonography of trauma (FAST) were negative. Mildly enlarged thyroid, diminished respiratory sounds on the left side, and bruise marks on the lower right limb were observed on physical examination. No Graves’ ophthalmopathy was observed. Tachycardiac atrial fibrillation was detected on electrocardiography. Mild cardiac dilatation and pulmonary congestion were observed on chest radiography. Enlarged thyroid and mild pulmonary contusion were noted on thoracoabdominal contrast computed tomography (CT). Fracture of the distal end of the right tibia was noted from lower limb radiography, but no fatal organ damage was observed. Blood tests indicated hyperthyroidism, with concentrations of thyroid stimulation hormone (TSH) <0.005 μIU/mL, Free T3 32.08 pg/mL, and Free T4 >7.77 ng/mL. Thyroid-associated antibodies were measured on the subsequent day: antithyrotropin receptor antibody (TRAb) was 37 IU/L (<2.0 IU/L); antithyroid peroxidase antibody (anti-TPO antibody) was ≥ 600 IU/mL; and antithyroglobulin antibody was 505 IU/mL. Thyroglobulin was not tested. Thyroid ultrasonography revealed increased blood flow in the bilateral lobes.

Disturbance of consciousness progressed rapidly after admission. The patient fell into a coma within 1 hour. Based on the clinical symptoms, the presence of hyperthyroidism, and thyroid ultrasonography, the patient was diagnosed with TS associated with Graves’ disease. Administration of potassium iodide, thiamazole, hydrocortisone (200 mg/day), and β blocker (propranolol 2 mg/day) was initiated. He was intubated because of respiratory failure and progressive disturbance of consciousness. High-output heart failure and pulmonary edema developed concomitantly; diuretics (hANP 0.025–0.03 γ), short-acting β blocker (landiolol 1–5 γ), and norepinephrine (0.1–1.6 γ) were administered to stabilize his circulatory condition. Apparent circulatory collapse was avoided during his whole clinical course.

**Figure 1:**
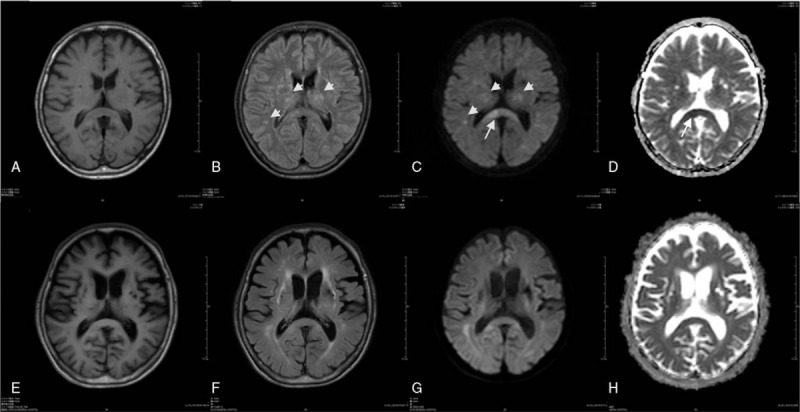
Brain MRI on the eighth hospital day. (A) T1 weighed image (T1WI); (B) fluid attenuated inversion recovery (FLAIR); (C) diffusion weighted image (DWI); (D) apparent diffusion coefficient (ADC) map) and 7 months after symptom onset; (E) T1WI; (F) FLAIR; (G) DWI; (H) ADC map. On acute phase, the splenium of the corpus callosum (SCC) lesion appeared DWI high intensity with low ADC (arrow). Lesions in bilateral thalami and cerebral white matter (arrowhead) showed high intensity in FLAIR and high intensity in DWI with low ADC. After 7 months, the SCC lesion disappeared but cerebral atrophy progressed. ADC = apparent diffusion coefficient, DWI = diffusion weighted image, FLAIR = fluid attenuated inversion recovery SCC = splenium of the corpus callosum, T1WI = T1 weighed image.

His thyroid function improved rapidly by virtue of these treatments, but disturbance of consciousness was protracted after withdrawal of sedative. No abnormality in electrolytes, blood glucose, liver function, or renal function that could explain disturbance of consciousness was detected from blood tests. No sign of worsening infection was found. Vitamins were supplied continually after admission. No episode of circulatory collapse that can induce hypoxic encephalopathy occurred during his clinical course. Electroencephalography was done on the fourth hospital day, eliciting no evidence of epileptic seizure. Lumbar puncture and cerebrospinal fluid test were performed on the sixth hospital day. The cell concentration was 1/μL. Protein was 31 mg/dL. Glucose was 63 mg/dL. Brain magnetic resonance imaging (MRI) was performed on the eighth hospital day, eliciting no finding suggesting brain contusion, diffuse axonal injury (DAI), or posterior reversible encephalopathy syndrome (PRES). In addition to these MRI findings, trauma-associated brain damage was regarded as unlikely because his consciousness was clear immediately after injury. However, multiple diffusion weighted image (DWI) high intensity with low ADC lesions was present in the splenium of the corpus callosum (SCC), bilateral thalami, and cerebral white matter (Fig. 1). These findings apparently indicate prolonged thyroid crisis-associated encephalopathy, although such a long clinical course is atypical for TS-related encephalopathy.

**Figure 2:**
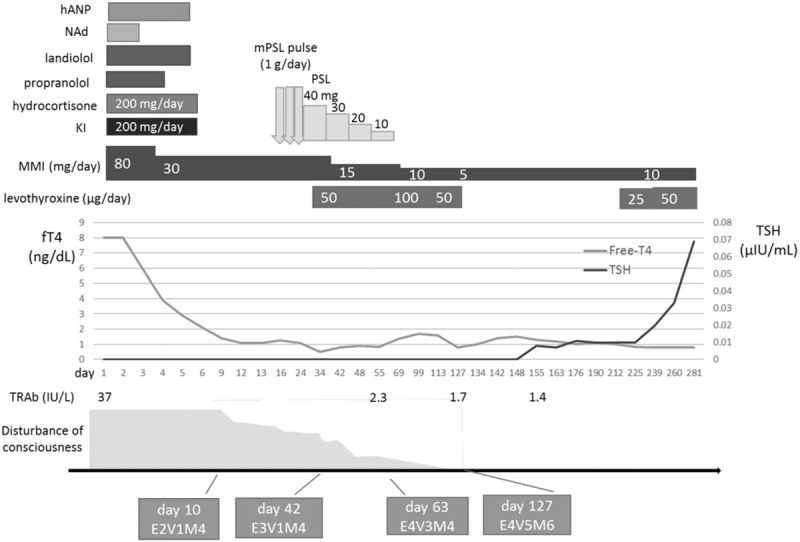
Treatment and clinical course of our case: hANP (human atrial natriuretic peptide); NAd, (noradrenalin); KI, (potassium iodine); and MMI, (thiamazole). human hANP = atrial natriuretic peptide, KI = potassium iodine, MMI = thiamazole, NAd = noradrenalin.

His GCS score had not improved to better than 7 (E2V1M4) during the next 2 weeks. Considering possible immune-related mechanism, methylprednisolone (mPSL) pulse therapy was started on the 22nd hospital day with subsequent oral corticosteroid therapy. His level of consciousness improved slowly. Finally, his GCS score reached 15 (E4V5M6) after 3 months (Fig. 2). He became able to walk with some assistance after 6 months. He was transferred to another hospital for rehabilitation. Moderate cognitive dysfunction persisted thereafter. Brain MRI performed after 7 months revealed that the abnormal lesion seen in the SCC had mostly disappeared, but global brain atrophy had progressed, especially in the cerebral cortex and deep gray matter (Fig. 1).

## 3 Discussion

This case was one of TS with protracted neurologic dysfunction. The clinical course of TS was severe, requiring endotracheal intubation and respiratory support, and multiple inotropic agents. Although critical circulatory collapse was avoided in his acute phase, his disturbance of consciousness was protracted even after normalization of the thyroid function, requiring 3 months for improvement. This point was a particularly atypical point of our case.

We first present discussion of the causes of his protracted consciousness disturbance. In contrast to our case, neurological dysfunction improves immediately after thyroid function improves in most TS cases. In these cases, thyroid hormone increases oxygen requirements by increasing the number of mitochondria.^[5]^ Also in such cases, relative hypoxic states for the brain can occur temporarily. However, irreversible hypoxic ischemic encephalopathy does not occur in most TS patients. Our case was atypical both as TS-related encephalopathy and hypoxic ischemic encephalopathy because the disturbance of consciousness persisted months after thyroid function normalization, but it was reversible. In addition, severe circulatory collapse that might induce systemic hypoxia was avoided in this case's clinical course.

On the brain MRI during his coma state, lesions with high intensity on DWI and low intensity in ADC were present in the SCC. Four earlier case reports describing TS with similar abnormal MRI findings were found from a search of PubMed and Japana Centra Revuo Medicina.^[6–9]^ Earlier reports have described that abnormal neurological manifestations were resolved within 2 weeks after normalization of the thyroid function by treatment, although our case required 3 months for recovery Similarly to those reported in earlier cases, the SCC lesion was reversible in our case. However, in our case, abnormal lesions found using brain MRI were not limited to the corpus callosum, but were instead scattered into other regions including bilateral tharami and cerebral white matter. Marchiafava–Bignami disease was regarded as a differential diagnosis based on the MRI findings. However, that diagnosis was also unlikely to be true considering that he had no evident history of long-term, heavy alcohol consumption. Moreover, his symptoms progressed rapidly after admission. Multiple reversible lesions other than SCC might be associated with protracted disturbance of consciousness.

Second, we assess the relation between systemic corticosteroid treatment and patient recovery. Hashimoto's encephalopathy, which is characterized by antithyroid antibody-positivity, presents subacute confusion or variable neurological manifestation. It is also known to cause cognitive dysfunction, neuropsychiatric symptoms, and disturbance of consciousness. Elevated anti-NH_2_-terminal of α-enolase (NAE) antibody is reported as a specific biomarker for Hashimoto's encephalopathy, but this antibody is found in only 68% patients of Hashimoto's encephalopathy.^[10]^ No definite diagnostic criteria for Hashimoto's encephalopathy exist. Thyroid hormones reportedly act on immune system cells.^[11,12]^ Actually, T3 has the function of promoting microglial migration and phagocytosis.^[13]^ Excess T3 in the central nervous system that can occur because of thyroid dysfunction is assumed to induce abnormal immunity in the central nervous system through these actions. These abnormal conditions of immune system are suggested as physiological mechanisms for Hashimoto encephalopathy. Therefore, it is reasonable to infer that TS induced a “Hashimoto's encephalopathy-like” condition. Because many cases of Hashimoto's encephalopathy are known to respond well to corticosteroid therapy,^[4]^ systemic corticosteroid administration is regarded as a treatment option in our protracted disturbance of consciousness case. However, we were unable to ascertain definitively whether corticosteroid therapy was truly effective in this case, or not.

Considering this case's clinical course, the long-term neurological outcome must be judged carefully even when disturbance of consciousness persists for a long time for severe TS with poor general condition and severe organ dysfunction. The disturbance of consciousness is reversible.

## 4 Conclusion

This report describes a case of TS with protracted disturbance of consciousness and abnormal brain MRI findings. Because this disturbance of consciousness is reversible with a month's clinical course, the long-term neurological outcome in TS patients must be judged carefully.
